# Replication of Influenza A Virus in Secondary Lymphatic Tissue Contributes to Innate Immune Activation

**DOI:** 10.3390/pathogens10050622

**Published:** 2021-05-19

**Authors:** Sarah-Kim Friedrich, Rosa Schmitz, Michael Bergerhausen, Judith Lang, Vikas Duhan, Cornelia Hardt, Matthias Tenbusch, Marco Prinz, Kenichi Asano, Hilal Bhat, Thamer A. Hamdan, Philipp Alexander Lang, Karl Sebastian Lang

**Affiliations:** 1 Institute of Immunology, University of Duisburg-Essen, Hufelandstrasse 55, 45147 Essen, Germany; sarah-kimfriedrich@gmx.net (S.-K.F.); Rosa.Schmitz@gmx.de (R.S.); bergerhausen@abalos-tx.com (M.B.); Judith.Lang@uk-essen.de (J.L.); vikas.duhan08@gmail.com (V.D.); Cornelia.Hardt@uk-essen.de (C.H.); bhathilal673@gmail.com (H.B.); 2 Institute of Clinical and Molecular Virology, University Hospital Erlangen, Friedrich-Alexander University Erlangen-Nürnberg, 91054 Erlangen, Germany; matthias.tenbusch@fau.de; 3 Institute of Neuropathology, Faculty of Medicine, University of Freiburg, 79106 Freiburg, Germany; marco.prinz@uniklinik-freiburg.de; 4 Centre for Integrative Biological Signalling Studies (CIBSS), University of Freiburg, 79106 Freiburg, Germany; 5 Centre for NeuroModulation (NeuroModBasics), University of Freiburg, 79106 Freiburg, Germany; 6 Signalling Research Centres BIOSS and CIBSS, University of Freiburg, 79106 Freiburg, Germany; 7 Laboratory of Immune Regulation, School of Life Science, Tokyo University of Pharmacy and Life Sciences, Tokyo 192-0392, Japan; asanok@toyaku.ac.jp; 8 Center for Molecular Medicine Cologne (CMMC), University Hospital Cologne, Robert Koch-Strasse 21, 50931 Köln, Germany; 9 Department of Medical Laboratories, Faculty of Health Sciences, American University of Madaba, Amman 11821, Jordan; 10 Institute of Molecular Medicine II, University of Düsseldorf, Universitätsstrasse 1, 40225 Düsseldorf, Germany; langp@uni-duesseldorf.de

**Keywords:** enforced viral replication, Influenza virus, innate immune activation

## Abstract

The replication of viruses in secondary lymphoid organs guarantees sufficient amounts of pattern-recognition receptor ligands and antigens to activate the innate and adaptive immune system. Viruses with broad cell tropism usually replicate in lymphoid organs; however, whether a virus with a narrow tropism relies on replication in the secondary lymphoid organs to activate the immune system remains not well studied. In this study, we used the artificial intravenous route of infection to determine whether Influenza A virus (IAV) replication can occur in secondary lymphatic organs (SLO) and whether such replication correlates with innate immune activation. Indeed, we found that IAV replicates in secondary lymphatic tissue. IAV replication was dependent on the expression of Sialic acid residues in antigen-presenting cells and on the expression of the interferon-inhibitor UBP43 (*Usp18*). The replication of IAV correlated with innate immune activation, resulting in IAV eradication. The genetic deletion of *Usp18* curbed IAV replication and limited innate immune activation. In conclusion, we found that IAV replicates in SLO, a mechanism which allows innate immune activation.

## 1. Introduction

Antigen-presenting cells (APC) are major initiators of the innate and adaptive immune response [1,2]. With their ability to express costimulatory molecules, APCs like dendritic cells (DCs) efficiently prime antigen-specific adaptive immune cells [2,3]. During virus infection, signaling via pattern recognition receptors (PRRs), such as Toll-like receptors (TLRs), or via retinoic acid inducible gene I (RIG-I), activate the PI3K-AKT-NF-κB pathway, which supplements antiviral activity in addition to type I interferons (IFN-I) [4,5]. In addition, TLR signaling induces IFN-I and results in the maturation and activation of DCs [6,7]. Antiviral effector programs in DCs target several stages of a virus life cycle, including viral genome replication, transcription and translation [8]. In addition, recent evidence highlights cholesterol metabolism as one major target of IFN-I induced antiviral activity [9].

The replication of a virus can enhance the amount of antigen and PRR-ligands, and therefore can be beneficial for immune activation [10,11,12]. During systemic infection with the vesicular stomatitis virus (VSV), CD169^+^ macrophages of the splenic marginal zone and the lymph node sinusoid can express the endogenous IFN-I blocker *Usp18,* thereby promoting virus replication and enhancing the antiviral immune response [11,13]. In line with this, during an infection with the lymphocytic choriomeningitis virus (LCMV), a *Usp18*-dependent replication in the APCs of the spleen and the lymph node is needed for efficient immune activation [12].

In contrast to LCMV and VSV, the Influenza A virus (IAV) has a narrower tropism, which usually leads to a highly specific replication of IAV in the lung epithelial cells [14]. Two major mechanisms contribute to this cellular tropism of IAV. First, the natural route of infection, which is via aerosols intra-nasally. Second, lung epithelial cells strongly express α2,3-sialic acid, which serves as an entry receptor for H1N1 IAV-types. Whether such entry mechanisms similarly exist in antigen-presenting cells and whether this contributes to the known strong innate immune activation during IAV infection remains mainly unknown [15].

Here, we use the intravenous route of infection to determine the role of IAV replication in secondary lymphatic organs. Indeed, we found that, during intravenous infection of IAV, early virus replication occurs mainly in the spleen. IAV replication occurred in CD11c-positive cells and is dependent on the expression of sialic acid as well as the interferon inhibitor UBP43 (*Usp18*). Attempts to limit IAV replication in secondary lymphatic tissue limited innate immune activation, suggesting that innate immune activation was a direct consequence of IAV replication.

## 2. Material and Methods

### 2.1. Mice

C57BL/6 mice were obtained from Taconic Bioscience. IFN-α/β receptor-deficient mice (*Ifnar^−/−^*)have been described elsewhere [16] and maintained on C57BL/6 background. *Usp18*^fl/fl^ mice were bred with mice expressing recombinase under the CAG promotor (CAG-cre) to generate CAG^+^ × *Usp18*^fl/fl^ and CAG^−^ × *Usp18*^fl/fl^ mice. *Usp18*^fl/fl^ mice were bred with mice expressing icre under the CD169 promotor (CD169-cre) to generate *CD169*-Cre^+/*ki*^ × *Usp18*^fl/fl^ and *CD169*-Cre^+/+^ × *Usp18*^fl/fl^ mice, as described in [17]. *Usp18*^fl/fl^ mice were generated by Marco Prinz [18]. CD169-cre mice were generated by Kenichi Asano [19]. CAG^+^ × *Usp18*^fl/fl^ mice were compared to CAG^−^ × *Usp18*^fl/fl^ littermate control mice. *CD169*-Cre^+/*ki*^ × *Usp18*^fl/fl^ mice were compared with *CD169*-Cre^+/+^ × *Usp18*^fl/fl^ littermate controls. For intravenous injection, the virus was injected into the tail vein at a volume of 100 μL. For intranasal injection, mice were narcotized with isoflurane and then IAV was given in a 20 μL dose, into the nose. Animal experiments were conducted with the authorization of Landesamt für Natur, Umwelt und Verbraucherschutz (LANUV) Nordrhein-Westfalen (Düsseldorf, Germany) and in accordance with the German law for animal protection.

### 2.2. Virus Quantification

The Influenza virus strain A/Puerto Rico/8/1934 H1N1 (IAV) was provided by M. Tenbusch. The IAV was propagated on Madin-Darby Canine Kidney II (MDCK II) cells. To analyze IAV titers, 11 serial 1:3 dilutions of samples were prepared. Every second dilution was transferred to pre-seeded, confluent MDCK II cells and overlaid with agarose solution in the presence of L-(tosylamido-2-phenyl) ethyl chloromethyl ketone (TPCK)-treated Trypsin (1 mg/mL, sigma-Aldrich) after 1 h of incubation. After 48–72 h, plaque-forming units were visualized by staining with crystal violet.

### 2.3. Reagents

Oseltamivir Phosphate was obtained from Sigma-Aldrich. Oseltamivir was administered orally twice a day at 0.1 mg/g one day prior to infection [20]. Sialidase was purchased from Roche. Mice were treated with 0.012 U Sialidase in phosphate buffered saline (PBS) i.p. The treatment was performed twice a day, starting one day prior to infection. The treatment was adjusted from the published protocol [21].

### 2.4. Immunofluorescense

Histological analysis was performed by preparing 8 µm thick sections from samples of snap frozen organs. IAV-Hemagglutinins were stained with anti-IAV-PR8 Hemagglutinin antibody (Sinobiological). Anti-CD169 (clone MOMA-1) was obtained from Bio-Rad. Anti-CD11c (clone N418) was obtained from eBioscience. Biotinylated Maackia Amurensis Lectin II (MAL II) was purchased from VectorLabs (B-1265-1) and visualized by a secondary Steptavidin-APC antibody staining (Biolegend, 405207).

Acetone (HoneyWell) fixation occurred for 10 min and non-specific binding site blocking was performed using two percent fetal calf serum (FCS, Gibco) in PBS for 15 min. Sections were incubated with antibodies diluted 1:100 in blocking buffer. Antibodies were incubated with the slides for 30–60 min in a humidified darkened chamber. Slides were covered with Fluorescence Mounting Medium (Dako) and image processing was performed using the Keyence BZ-9000 microscope.

### 2.5. Flow Cytometry

Surface antigens on splenocytes were stained using anti-CD169 (clone MOMA-1), anti-CD11c (clone N418), anti-CD8a (clone 53-6.7, eBioscience), anti-CD4 (clone L3T4, eBioscience), anti-F4/80 (clone BM8, eBioscience), and fixable viability dye (eBioscience). Cells were analyzed using the flow cytometer LSR Fortessa (Becton Dickinson).

### 2.6. Intracellular Cytokine and IAV Staining

IAV-specific interferon gamma (IFNγ)-producing T cells were analyzed by intracellular cytokine staining, adapted from the protocol described elsewhere [22]. Briefly, mouse splenocytes were incubated overnight with IAV peptide (YTDIEMNRLGK, Anaspec) at 0,1 µg/mL in the presence of Brefeldin A (Sigma-Aldrich). Surface epitopes were subsequently stained with anti-CD8a (clone 53-6.7) and anti-CD4 (clone L3T4) antibodies. After fixation and permeabilization, intracellular IFNγ was stained with anti-IFNγ antibody (clone XMG1.2) purchased at eBioscience.

IAV^+^ cells were analyzed via intracellular IAV staining. Splenocytes of infected or naive mice were stained for surface antigens as described. Cells were fixed and permeabilized and subsequently stained with anti-IAV-PR8 Hemagglutinin antibody, followed by a secondary antibody (Goat anti-Rabbit, ThermoFisher).

Cells were analyzed using the flow cytometer LSR Fortessa (Becton Dickinson).

### 2.7. RNA Extraction, cDNA Synthesis and qRT-PCR

Whole RNA extraction was performed with Trizol Reagent (Invitrogen) according to the manufacturer’s protocol. Synthesis of cDNA was performed with a QuantiTect Reverse Transcription kit (Qiagen). Isopropanol (Sigma) precipitated RNA was washed twice with 70% ethanol (Sigma). A total of 400 ng RNA was synthesized into cDNA. The gene expressions of *Ifna2* (QT00253092), *Ifna4* (QT01774353), *Ifna5* (QT00327656) and *Ifnb1* (*QT00249662*), were analyzed with primers obtained from Qiagen and normalized to GAPDH (QT01658692). IAV expression was analyzed by a primer sequence made in house (5′CTTCTAACCGAGGTCGAAACG3′,5′ GGGCATTTTGGACAAAG/TCGTCTA 3′).

### 2.8. ELISA

Mouse interferon alpha ELISA was obtained from Invitrogen and performed according to the manufacturer’s protocol.

### 2.9. Statistical Analysis

If not mentioned otherwise, data are expressed as the arithmetic mean ± SEM and Student’s *t*-test was used to detect statistically significant differences (one- or two-tailed). P values of 0.05 or less were considered statistically significant. Statistical analyses and graphical representations were computed with Graph Pad Prism software version 6 (Graph Pad, La Jolla, CA, USA).

## 3. Results

### 3.1. IAV Replicates in the Spleen during Systemic Infection

**Figure 1:**
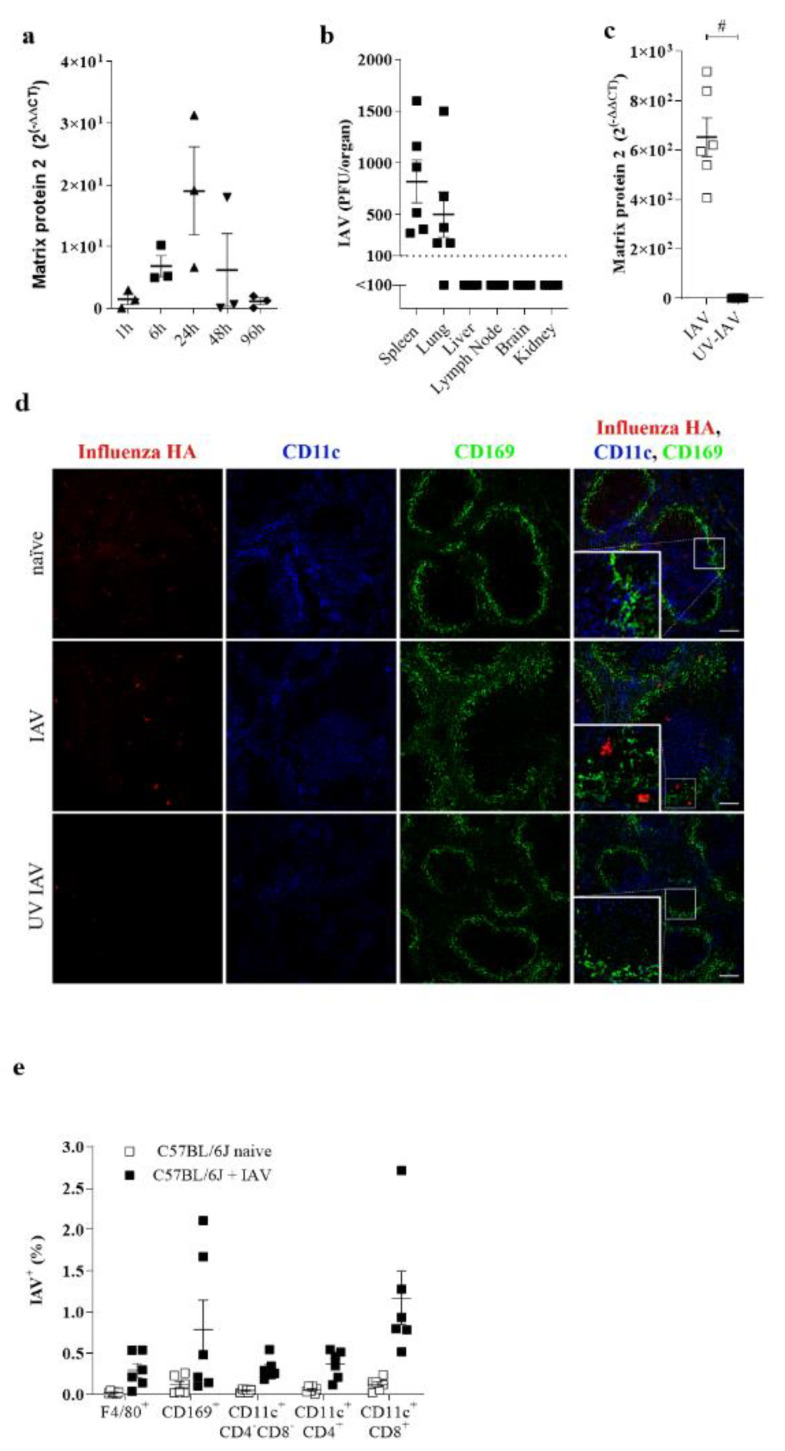
IAV replicates in the spleen during systemic infection. (**a**) Relative expression of IAV Matrix Protein 2 in spleens of C57BL/6 mice infected with 10^6^ PFU of IAV i.v. normalized to naïve spleen values (*n* = 3). (**b**) Viral titers of indicated organs in C57BL/6 mice 24 h p.i. with 10^7^ PFU IAV i.v (*n* = 6). (**c**) Quantitative analysis of Matrix Protein 2 expression via rtPCR of mice normalized to naïve spleen values (*n* = 6). (**d**) Immunofluorescense of spleen sections of naïve C57BL/6 or C57BL/6 i.v. infected with 10^7^ PFU live or UV inactivated IAV stained for CD169 (green), CD11c (blue) and IAV HA (red). Figure is representative of 2 independent experiments (*n* = 6). (**e**) Intracellular staining of IAV^+^ cells of indicated myeloid cells from naïve C57BL/6 mice (black), mice infected with 10^7^ PFU IAV i.v. (white) or mice pretreated with Oseltamivir (**c**, dotted white) infected with 10^7^ PFU IAV i.v. 24 h p.i. (*n* = 6). *# p* < 0.0001 (Student’s *t*-test, **a**–**c**). Data are representative of two (**a**–**c**,**e**) experiments (mean ± s.e.m. (**a**–**c**)). The scale bar represents 100 μm.

To analyze the role of lymphoid organs during influenza infection without the direct influence of the IAV replication in the lung tissue, we considered that the intravenous infection of IAV would give insights into the capacity of innate immune cells in replicating IAV. About 6 h after systemic infection, IAV was detectable in the spleen (Figure 1a). Expression increased over time with a maximum propagation demonstrated after 24 h of infection (Figure 1a). Intriguingly, IAV replication along the intravenous route was higher in the spleen than the lung tissue, considering that, during this route of infection, innate immune cells were the first cells to replicate IAV (Figure 1b). To prove that detected RNA was due to active replication in the spleen, we injected mice with UV-inactivated IAV. Treatment with UV-IAV showed limited IAV RNA accumulation in the spleen, suggesting that, indeed, the virus needed to replicate for RNA levels to be detectable (Figure 1c). To obtain insight into which cells the IAV replicates, we performed a histological analysis of the splenic sections from IAV infected mice. We found that IAV propagates mainly in the marginal zone and the lymph follicle (Figure 1d). Some infected cells colocalized with CD11c or CD169 (Figure 1d). Flow cytometric analysis revealed that mainly CD169^+^ macrophages and CD8^+^ CD11c^+^ DCs were stained positive for IAV (Figure 1e). In conclusion, we found that IAV replicates specifically in splenic marginal zone macrophages and/or antigen-presenting cells.

### 3.2. Replication of IAV in the Spleen Correlated with IFN-I Induction

**Figure 2:**
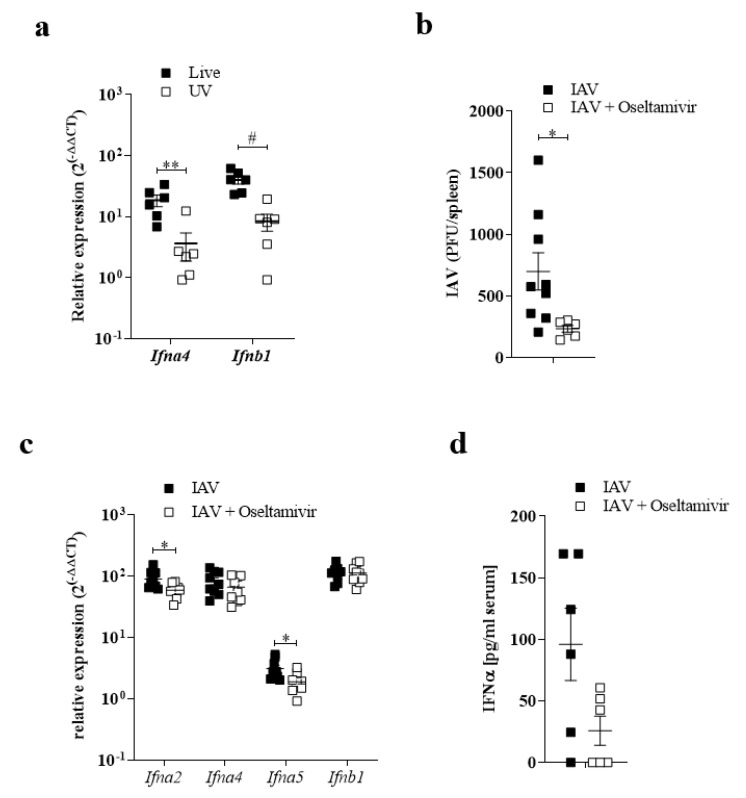
Replication of IAV in the spleen correlated with IFN-I induction. (**a**) Relative expression of *Ifnα4* and *Ifnβ1* of C57BL/6 mice i.v. infected with 10^7^ PFU live or UV inactivated IAV (*n* = 6). (**b**) IAV titers of spleen samples obtained from mice pretreated with and without Oseltamivir and infected with 10^7^ PFU IAV i.v. 24 h p.i. (*n* = 5–9). (**c**) Relative expression of *Ifnα2, Ifnα4, Ifnα5* and *Ifnβ1* of spleen samples obtained from mice pretreated with and without Oseltamivir and infected with 10^7^ PFU IAV i.v. 24 h p.i. (*n* = 9). (**d**) IFN*α* levels in serum from mice pretreated with and without Oseltamivir and infected with 10^7^ PFU IAV i.v. 24 h p.i. (*n* = 6): * *p* < 0.05; ** *p* < 0.01; # *p* < 0.0001 (Student’s *t*-test, **a**–**d**). Data are representative of two (**a**,**b**) or three (**c**,**d**) independent experiments (mean ± s.e.m. (**a**–**d**)).

Remarkably, we found that IAV could replicate in the spleen after systemic infection. Next, we wondered whether this replication was involved in innate immune activation in the spleen. With this aim, we first compared the immune activation capacity of a live virus and a UV-inactivated IAV. An injection of UV-IAV limited the induction of Ifna4 and Ifnb1 expression in splenic tissues (Figure 2a). Further, we treated mice with the antiviral drug Oseltamivir [23]. Treatment with the Oseltamivir limited the replication of IAV in the spleen (Figure 2b). We found limited induction of IFN-I in the spleen as well as limited systemic levels of IFN-I (Figure 2c,d). Taken together, we conclude that the replication of IAV in the spleen can be attributed to innate immune activation.

### 3.3. Sialic Acid Residues Are Essential for IAV Uptake

**Figure 3:**
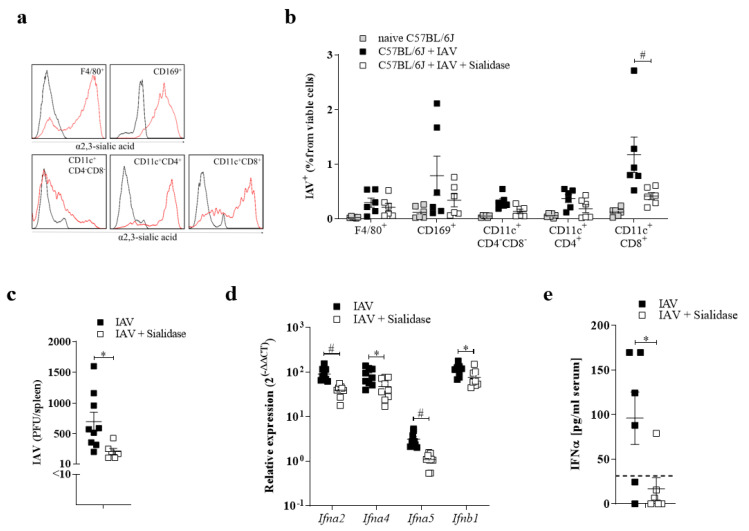
Sialic acid residues are essential for IAV replication in the spleen. (a) Representative histograms of myeloid cell populations binding to secondary antibody control (black line) or MAL II lectin (α2,3-SA). One mouse from two independent experiments (*n* = 3 mice per experiment) is shown. (**b**) Intracellular staining of IAV^+^ cells of indicated myeloid cells from naïve C57BL/6 mice (black), mice infected with 10^7^ PFU IAV i.v. (white) or mice pretreated with Sialidase i.v. and infected with 10^7^ PFU IAV i.v. 24 h p.i. (*n* = 6). (**c**) IAV titers of spleen samples from mice with and without pretreatment with Sialidase i.p. and then infected with 10^7^ PFU IAV i.v. 24 h p.i. (**d**,**e**) Relative expression of *Ifnα2, Ifnα4, Ifnα5* and *Ifnβ1* (*n* = 6–9). (**d**) or serum IFN*α* levels (*n* = 9). (**e**) mice with and without pre-treatment with Sialidase and then infected with 10^7^ PFU IAV i.v. 24 h p.i. (*n* = 6). Dotted line represents the detection level for IFN*α* ELISA. * *p* < 0.05, (Student’s *t*-test, **c***–***e**). Data are representative of two (**a**–**c**) or three (**d**,**e**) independent experiments (mean ± s.e.m. (**a,c**).

Several entry and host factors are known to promote IAV replication [24]. In addition to this, unknown factors might contribute to the propagation of IAV. Especially, infection of antigen-presenting cells seems to not always be strictly dependent on one entry receptor [25]. α2,3- sialic acid (SA) residues bind to Influenza A H1N1 PR/8 strain and thereby promote infection of the cells [26,27,28]. To see whether SA is involved in IAV uptake after i.v. infection, we analyzed α2,3- SA expression in myeloid cell populations in the spleen. The highest expression of α2,3- SA was detected on F4/80^+^ cells, CD169^+^ cells as well as CD11c^+^ dendritic cell subsets; CD11c^+^CD8^+^ and CD11c^+^CD4^+^ cells (Figure 3a). 

Next, we wanted to examine whether SA residues on myeloid cells contribute to IAV uptake. To do so, we treated mice intraperitoneally with Sialidase and infected them intravenously with IAV. Using this mode of treatment, we were able to analyze the direct impact of Sialidase and IAV on myeloid cells. Treatment with Sialidase limited the infection of antigen-presenting cells in the spleen, as demonstrated by the lower viral titer upon treatment. Notably, CD11c^+^CD8^+^ DCs showed a severe reduction in IAV infection (Figure 3b,c). These findings demonstrate that SA residues on cells are necessary to take up IAV after i.v. infection. Next, we assumed that limited uptake and replication of IAV in antigen-presenting cells limits the systemic type I interferon (IFN-I) response. Indeed, IFN-I responses were reduced in Sialidase treated mice, as documented by diminished IFNI-I expressing genes and IFN-I serum levels (Figure 3d,e). Collectively, we demonstrated that uptake of IAV in myeloid cells via SA is essential for activation of the innate immune system.

### 3.4. Usp18 Promotes Replication of IAV in Antigen-Presenting Cells

**Figure 4:**
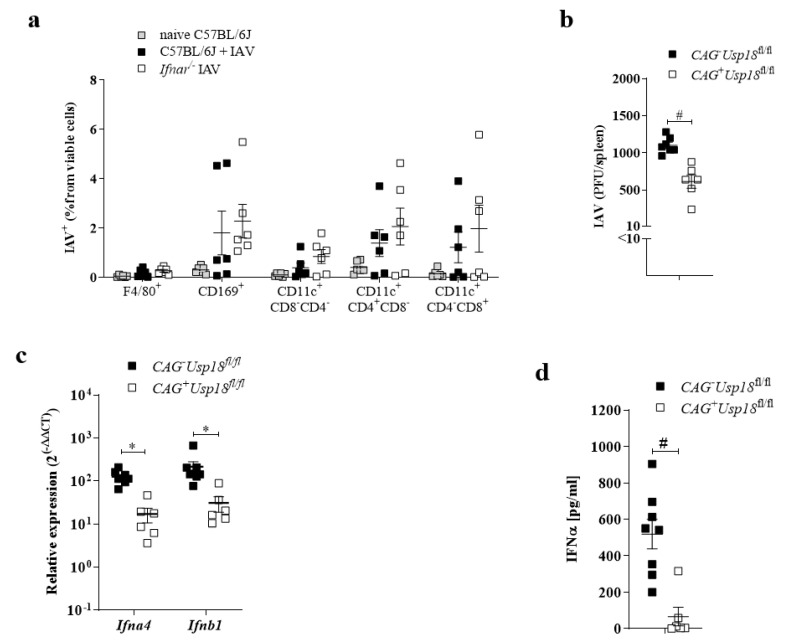
*Usp18* promotes replication of IAV in antigen-presenting cells. (**a**) Intracellular staining of IAV^+^ cells of indicated myeloid cells from naïve C57BL/6 mice (black) or WT or *Ifnar^−/−^* mice, infected with 10^7^ PFU IAV i.v. analyzed 24 h p.i. (*n* = 6). (**b**) IAV titers from spleens of Cag-Cre^+^*Usp18*^fl/fl^ and Cag-Cre*^−^Usp18*^fl/fl^ mice infected with 10^7^ PFU IAV i.v. after 24 h (*n* = 6–7). **c+d** Relative *Ifnα4* and *Ifnβ1* expression in the spleen (**c**) -Serum IFNα levels. (**d**) Samples derived from CAG^+^*Usp18*^fl/fl^ and CAG*^−^Usp18*^fl/fl^ mice infected with 10^7^ PFU IAV i.v. after 24 h (*n* = 6–8). * *p* < 0.05, and # *p* < 0.001 (Student’s *t*-test, **c**,**d**). Data are representative of two (**a**–**d**) experiments (mean ± s.e.m. (**a**–**d**)).

We identified UBP43 (*Usp18*), an endogenous IFN-I blocker, as one important factor which promotes replication of viruses in antigen-presenting cells. Indeed, replication of VSV, the EBOLA vaccine virus (VSV-EBOV), LCMV and HIV [10,11,12,17,29,30,31] depend strongly on the expression of *Usp18* in myeloid cells. Mechanistically, *Usp18* strongly limited the type I interferon response and thereby facilitated replication of the virus. We hypothesized that *Usp18* in dendritic cells similarly influenced IAV replication. We analyzed the role of IFN-I on early replication of IAV in APCs. Indeed, when we infected IFNAR-deficient mice with IAV, we detected no obvious differences in IAV replication between WT and *Ifnar^−/−^* CD169^+^ macrophages, CD4^+^CD11c^+^ or CD8^+^CD11c^+^ dendritic cells (Figure 4a). This suggests that the role of IFN-I in controlling IAV was limited in these cell types, which hints at the expression of endogenous IFN-I blocker. To address whether replication of IAV in the spleen is dependent on *Usp18*, we infected Tamoxifen-treated Cag-Cre^+^*Usp18*^fl/fl^ (*CAG*^+^ *Usp18*^fl/fl^)and Cag-Cre^−^*Usp18*^fl/fl^ (*CAG^−^Usp18*^fl/fl^)mice with IAV and analyzed viral replication. Lack of *Usp18* limited IAV replication in the spleen (Figure 4b). To examine the impact of *Usp18* deficiency on IFN-I levels and subsequent innate immune activation, we infected Tamoxifen-treated Cag-Cre^+^*Usp18*^fl/fl^ and Cag-Cre*^−^Usp18*^fl/fl^ mice with IAV and measured the IFN-I levels and found that the lack of *Usp18* insufficiently restrains IFN production (Figure 4c,d). Together, *Usp18* insufficiency curbed IFN-I expression in response to IAV infection.

### 3.5. Usp18 Dependent Replication Controls Virus Propagation in the Lung

**Figure 5:**
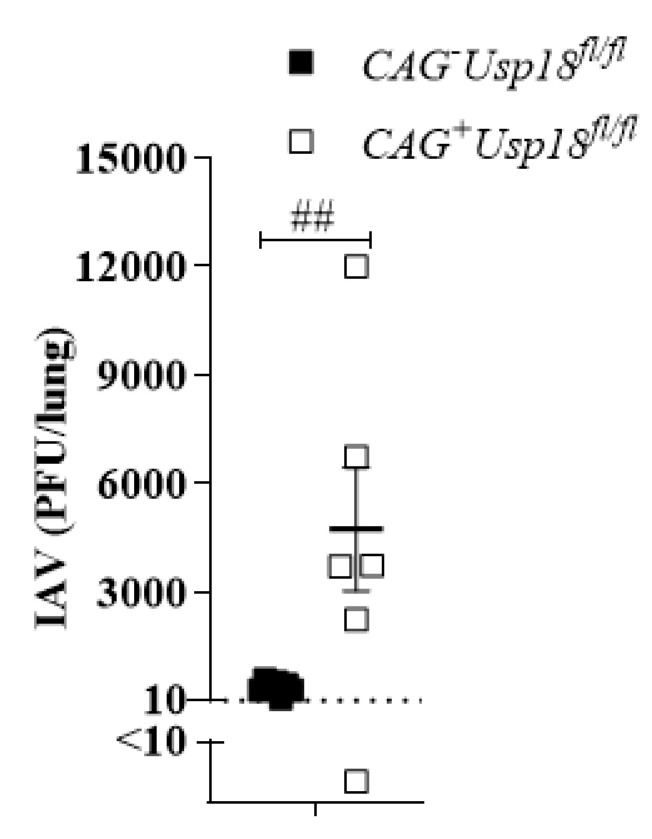
*Usp18*-dependent replication controls virus propagation in the lung. IAV titers from the lung of Cag-Cre^+^*Usp18*^fl/fl^ and Cag-Cre*^−^Usp18*^fl/fl^ mice infected with 10^7^ PFU IAV i.n. after 24 h. ## *p* < 0.0001 (*n* = 6–7). Data are representative of two experiments (mean ± S.E.M.).

We found that IAV can replicate in lymphatic tissue and this replication correlates with innate immune activation. Next, we aimed to analyze whether this mechanism was also important for local virus control. To that end, we intranasally infected Tamoxifen-treated Cag-Cre^+^*Usp18*^fl/fl^ and Cag-Cre*^−^Usp18*^fl/fl^ mice with IAV and then analyzed virus replication in the lung. Indeed, we detected accelerated replication of IAV, suggesting that the lack of IFN-I might contribute to limited viral control (Figure 5). Together, these data show that *Usp18* is a molecule key modulator which accelerates IAV replication in the spleen and thereby enhances IFN-I induction.

## 4. Discussion

Virus replication in immune cells is a key process for immune activation. Even during vaccination with attenuated pathogens, the replication of a virus is required, as in the cases of measles, rubella, chicken pox, yellow fever and mumps [32]. Here, we found that also during IAV infection, replication of the virus in APCs can be beneficial for innate immune activation.

The replication of a virus is mainly determined by its cellular tropism. The vesicular stomatitis virus (VSV) and the lymphocytic choriomeningitis virus (LCMV) can both infect a broad range of cell types. However, in the presence of type I interferon (IFN-I) replication can mainly be detected in secondary lymphatic organs [10,11,33]. This replication is explained by the expression of *Usp18* in APCs in the spleen and the lymph node, which allows replication despite high IFN-I levels. In contrast to VSV and LCMV, IAV has a narrow tropism, and is mainly replicated within lung tissue. Here, we show that during systemic infection with IAV, replication is also detected in APCs in the spleen.

α2,3- sialic acids are mainly expressed in lung tissue (Supplementary Figure S1) and this expression is one factor that promotes the lung specificity [34]. Surprisingly, we also found some expression of α2,3- sialic acid on myeloid cells in the spleen. The strongest expression was seen in CD8^+^ CD11c^+^ dendritic cells. The expression of α2,3-sialic acid correlated with the infection rate of IAV. Treatment with Sialidase limited IAV replication in APCs, suggesting that, indeed, the expression of α2,3-sialic acid contributed to IAV replication. As well as sialic acid, the expression of *Usp18* also contributed to IAV replication in the spleen. This could be explained by the IFN-I inhibiting activity of the *Usp18*. In earlier studies, it was demonstrated that the local draining lymph node is involved in the immune activation against IAV [35,36,37,38,39,40,41,42,43]. Furthermore, lymphotoxine-alpha-deficient mice, which were shown to have an impaired replication of the virus in the lymphatic tissue [10,12], do have an enhanced susceptibility to local IAV infection [44]. In line with this, we found that the lack of *Usp18*, which limits the replication of a virus in secondary lymphatic organs and/or immune cells, leads to enhanced susceptibility during intranasal infection.

We found that attempts to suppress the replication of IAV in the spleen limited the innate immune activation. Therefore, we consider it likely that the early replication of IAV in lymphoid organs contributes to antiviral activity. Indeed, mice which lack *Usp18* showed accelerated replication of IAV in the lung, suggesting that innate control is limited there. While we consider that early replication was mainly responsible for defective innate immune activation, other functions of *Usp18* might contribute to IAV control [29]. In conclusion, we found that IAV can replicate in the spleen, and thereby activate the innate immune response.

## Data Availability

Data is contained within the article or Supplementary Material.

## Supplementary Materials

The following are available online at https://www.mdpi.com/article/10.3390/pathogens10050622/s1, Figure S1: Expression of Sialic acid on different organs.
