# Supplementary material for: Replication of Influenza A Virus in Secondary Lymphatic Tissue Contributes to Innate Immune Activation
Source: Pathogens. 2021 May 19;10(5):622. doi: 10.3390/pathogens10050622 (PMC8160763; doi:10.3390/pathogens10050622)
Supplement: Supplementary file 1 [file pathogens-10-00622-s001.zip › pathogens-1102408-supplementary.pdf]

pathogens

MDPI

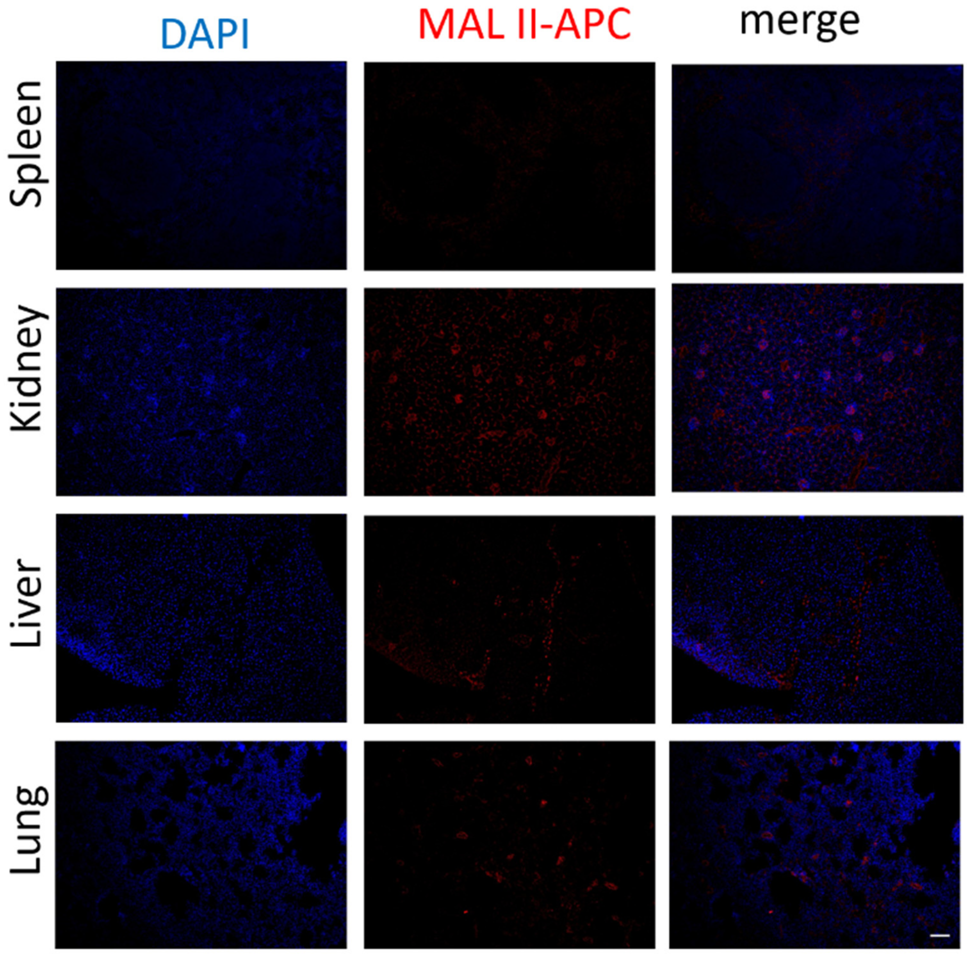

Supplementary Figure S1. Expression of Sialic acid on different organs. Representative immunofluorescence of spleen, kidney, liver and lung histological sections from B6/7J murine models stained for sialic acid. One slide, representative of four, is shown. Scale bar, 100 µm.
